# Community and Academic Synergy for Cancer Survivorship Care Delivery Enhancement (Project CASCADE): a study protocol for a pragmatic, stepped-wedge, cluster-randomized trial in Texas primary care community health centers

**DOI:** 10.1186/s12875-025-03108-1

**Published:** 2025-12-27

**Authors:** Mary-Louise E. Millett, Lauren Q. Malthaner, Katharine McCallister, Derek W. Craig, Rebecca Eary, Melissa A. Valerio-Shewmaker, L. Aubree Shay, Suja S. Rajan, Hilary Y. Ma, Samiran Ghosh, Benjamin F. Crabtree, Simon J. Craddock Lee, Bijal A. Balasubramanian

**Affiliations:** 1 Institute for Implementation Science, The University of Texas Health Science Center at Houston (UTHealth Houston), Houston, TX USA; 2 Department of Health Promotion and Behavioral Sciences, UTHealth Houston School of Public Health, Houston, TX USA; 3 UTHealth Houston School of Public Health in Dallas, Dallas, TX USA; 4 Department of Family and Community Medicine, University of Texas Southwestern Medical Center, Dallas, TX USA; 5 Department of Management, Policy and Community Health, UTHealth Houston School of Public Health, Houston, TX USA; 6 Department of General Oncology, Division of Cancer Medicine, The University of Texas MD Anderson Cancer Center, Houston, TX USA; 7 Department of Health Disparities Research, Division of Cancer Prevention & Population Sciences, The University of Texas MD Anderson Cancer Center, Houston, TX USA; 8 Department of Biostatistics and Data Science, UTHealth Houston School of Public Health, Houston, TX USA; 9 Department of Family Medicine and Community Health, Research Division, Rutgers Robert Wood Johnson Medical School, New Brunswick, NJ USA; 10 Department of Population Health, School of Medicine, Medical Center, University of Kansas, Kansas City, KS USA; 11 University of Kansas Cancer Center, Kansas City, KS USA; 12 Department of Epidemiology, UTHealth Houston School of Public Health in Dallas, Dallas, TX USA

**Keywords:** Cancer survivorship, Care delivery, Primary care, Community health centers

## Abstract

**Background:**

In the United States, over 18 million individuals are living with cancer. The majority of these cancer survivors also manage other chronic conditions and receive care from multiple specialists, including oncology, cardiology, and primary care clinicians. However, it remains unclear who holds primarily responsibility for coordinating their care across specialties. Because of its generalist nature, primary care is uniquely suited to deliver whole-person and coordinated care for all conditions for cancer survivors. However, primary care teams experience many challenges delivering high-quality survivorship care. While integrating care for all conditions including cancer is a core principle of high-quality primary care, few survivorship care delivery interventions have been developed and tested among patients with a history of cancer in primary care panels of community health centers (CHCs). These under- and uninsured cancer survivors experience disproportionately worse health outcomes and often rely solely on CHCs for consistent health care.

**Methods:**

*Community and Academic Synergy for Cancer Survivorship Care Delivery Enhancement* (Project CASCADE) is a theory-driven pragmatic hybrid trial testing implementation and effectiveness of a multi-component primary care-based survivorship care delivery intervention among 8 Texas CHC sites. The specific aims are: Aim 1: Implement a system-level cancer survivorship care delivery intervention in partnership with CHC clinicians, patients, and community representatives, which includes: (1) primary care clinician training in cancer survivorship care through provider-to-provider tele-mentoring, (2) identification and tracking of survivors by modifying existing clinic workflows, and (3) coordinating survivors’ care by designating a care coordinator champion. Practice facilitation and stakeholder engagement strategies will support intervention implementation; Aim 2: Test the effectiveness of the intervention on patient (screening for second primary cancers) and clinician outcomes (clinician knowledge of and confidence in survivorship care) using a stepped-wedge clinic-randomized design; and Aim 3: Evaluate implementation using a mixed-methods approach guided by the Practice Change Model. We will utilize electronic health record (EHR), survey, interview, and observation data to assess effectiveness and implementation outcomes.

**Discussion:**

Findings will inform a scalable, generalist primary care-based survivorship care model to enhance care delivery and outcomes in CHCs serving vulnerable populations. This study represents a critical step toward addressing gaps in cancer survivorship research and achieving equitable care for all survivors.

**Trial registration:**

This study is registered under clinical trial registration number NCT06883838; 2025–03-12.

**Supplementary Information:**

The online version contains supplementary material available at 10.1186/s12875-025-03108-1.

## Introduction

The US has over 18 million cancer survivors, projected to reach 22 million by 2030 [1]. Following the National Cancer Institute (NCI) and the American Cancer Society, “cancer survivors” are persons diagnosed with cancer no matter where they are in the course of their disease [2, 3]. The seminal Institute of Medicine Reports on “Delivering High Quality Cancer Care” and “Lost in Transition – From Cancer Patient to Cancer Survivor” published almost 18 years ago highlighted the complex healthcare needs of survivors and the challenges delivering coordinated healthcare [4, 5], 70% of cancer survivors also have other chronic conditions [6, 7] requiring management across multiple clinicians including oncologists, cardiologists, and primary care physicians. In 2025, we still lack effective and disseminatable solutions for optimizing delivery of high-quality survivorship care.

Most survivorship care models are based within academic health systems and cancer centers [8–12] and have shown mixed results of effectiveness and impact [13–15]. The growing population of cancer survivors, coupled with a projected shortage of oncology specialists [16], highlights the potential lack of sustainability of a survivorship care model led by oncology alone. Emerging primary care-based models, demonstrate a viable alternative [17–19]. Many cancer survivorship care services resemble care provided for chronic diseases such as diabetes, hypertension, and heart disease such as identifying and reducing risks related to sequelae of chronic conditions and its treatments, promoting healthy behaviors, and addressing behavioral health issues. Primary care practices have the care delivery processes, tools, and clinic staff for tracking and managing patients with multiple chronic conditions and complex health care needs, including sequelae of cancer and its treatments. Therefore, primary care is well suited to deliver whole person and coordinated survivorship care to survivors.

However, there are barriers to leveraging this capacity [20]. Primary care clinicians (PCCs) often do not feel equipped to care for survivor needs [21], lack knowledge about survivorship care guidelines [22–25], and need more experience recognizing and managing the adverse effects of cancer treatments [26]. In addition, there is a perception that cancer prevalence is low in primary care panels [27]. A study of 431 community health centers (CHCs) noted that 3% of adult patients had cancer documented in their electronic health record (EHR) [28]; this is likely an underestimate [28] because of inconsistent documentation. Our chart audit study in two CHC clinics identified 6.5% patients with ≥ 2 chronic conditions had cancer [29].

To address these gaps, *Community and Academic Synergy for Cancer Survivorship Care Delivery Enhancement* (Project CASCADE), a pragmatic, type 2 hybrid implementation-effectiveness trial in Texas-based primary care community health center (CHC) clinics, aims to:

1. Implement a system-level cancer survivorship care delivery intervention in partnership with CHC clinicians, patients, and community representatives
2. Test effectiveness of the intervention to improve patient and clinician outcomes using a stepped- wedge cluster-randomized trial design
3. Evaluate implementation of the intervention using an iterative, concurrent mixed-methods approach guided by the Practice Change Model (PCM)

Accomplishing these aims will enhance the capacity of safety-net primary care teams to manage cancer survivorship in primary care, enabling survivors to receive coordinated, whole-person care.

## Methods

This study protocol adheres to The Standard Protocol Items: Recommendations for Interventional Trials (SPIRIT) guidelines (see supplementary file 1) [30]. Study results will be reported in adherence to the Consolidated Standards of Reporting Trials (CONSORT) guidelines [31] and the Standards for Reporting Implementation Studies (StaRI) guidelines [32]. In addition to publications, we plan to present results at scientific conferences and local community organization meetings.

### Study design

**Fig. 1:**
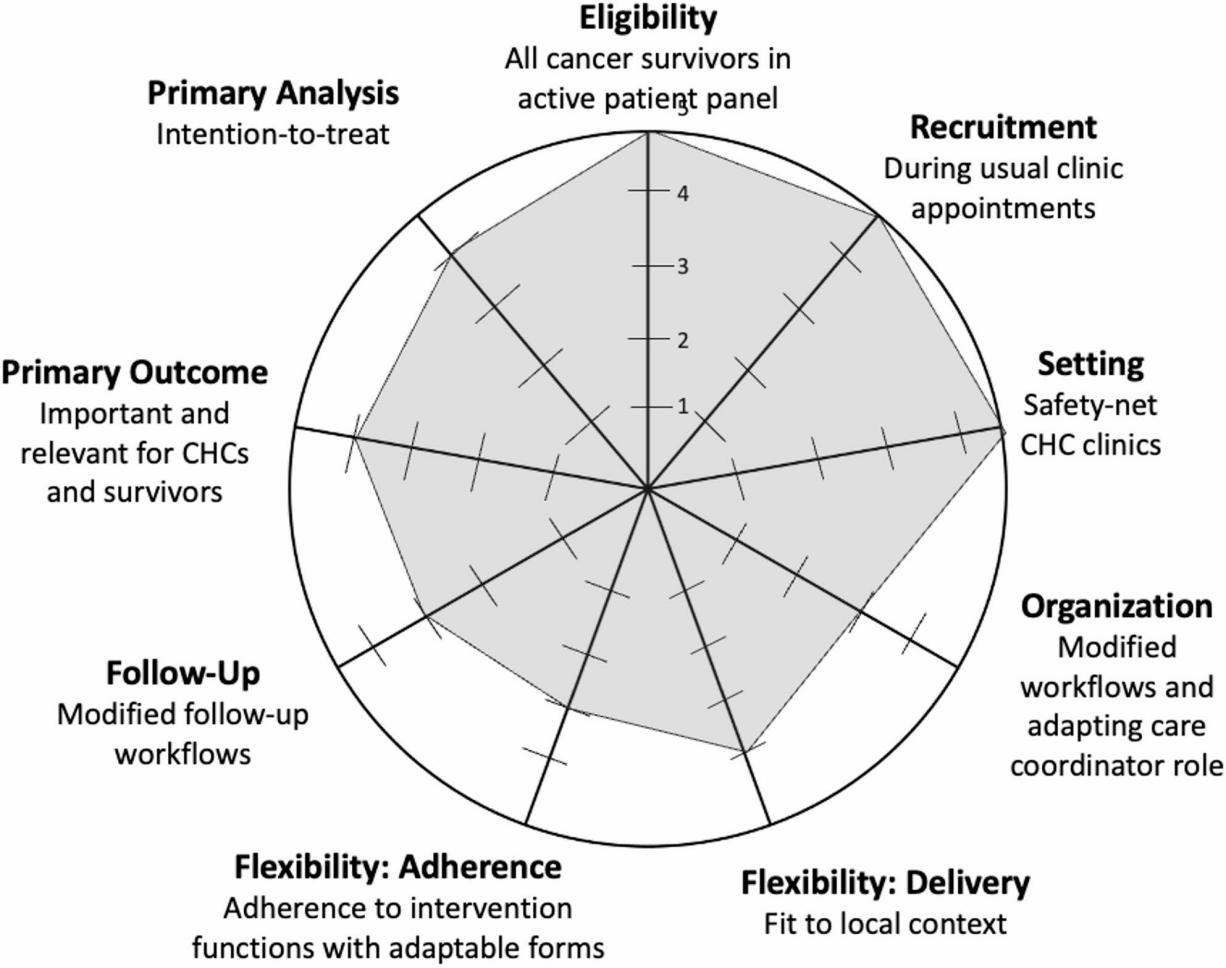
Use of PRECIS-2 in Project CASCADE

Project CASCADE is a pragmatic, Type II hybrid implementation-effectiveness trial using a cross-sectional, stepped wedge design coupled with a mixed-methods implementation evaluation. A stepped-wedge design is a type of constrained cluster-randomized trial in which groups (or clusters) of participants are sequentially randomized from a control condition to an intervention condition over multiple time periods [33, 34]. The key constraint is that randomization occurs only in one direction—from control to intervention condition. In this design, each cluster ultimately receives the intervention, and because data are collected over time, each cluster effectively serves as its own control. A Type II hybrid approach is ideal for this study as both intervention implementation and effectiveness are evaluated and have related study outcomes [35]. Stepped wedge designs are equitable by allowing for all clinics to receive the intervention [34, 36] at some point of time. In addition, we used the Pragmatic Explanatory Continuum Indicator Summary (PRECIS-2) to describe the parameters by which our study is pragmatic (Fig. 1) [37].

### Setting

10 clinics spanning five CHC systems have agreed to participate in this study*.* These include FQHCs and community-oriented primary care clinics affiliated with county safety-net systems (Table 1). Per power estimations, only 8 clinics are needed to test effectiveness. We selected CHCs with a high proportion of minority and under/uninsured individuals. In addition, the CHCs vary in size (5–15 clinicians) and geographic location (urban–rural continuum), allowing us to examine contextual factors influencing effectiveness and implementation [38, 39].

**Table 1:** Patient characteristics of partner CHCs

Community Health Center *(Partner Clinics)*	% Racial/Ethnic Minorities	% Uninsured/Medicaid	Estimated # Survivors seen per year per clinic*
CHC1 (2 clinics)	83%	53%	139
CHC2 (2 clinics)	98%	58%	106
CHC3 (2 clinics)	92%	77%	176
CHC4 (2 clinics)	91%	77%	607
CHC5 (2 clinics)	93%	88%	1008

^*^Estimated prevalence of 6.5% based on preliminary data [29]

### Participants

This system-level intervention will target clinic and clinic teams; patients will receive the intervention passively as part of the care they receive from their primary care teams. Eligible clinic team members include all employed clinicians (MD, DO, PA, NP) and clinic staff (e.g., social worker, medical assistant, care coordinator) of the participating CHC clinics. Eligible patients include current CHC patients that are at least 18 years old with a history of any type of cancer. This is an important pragmatic feature due to the strength of generalist primary care in prioritizing and delivering care for all conditions. An intervention focused on a single cancer is unlikely to be fully integrated into usual practice in community settings [27].

### Randomization procedures

**Fig. 2:**
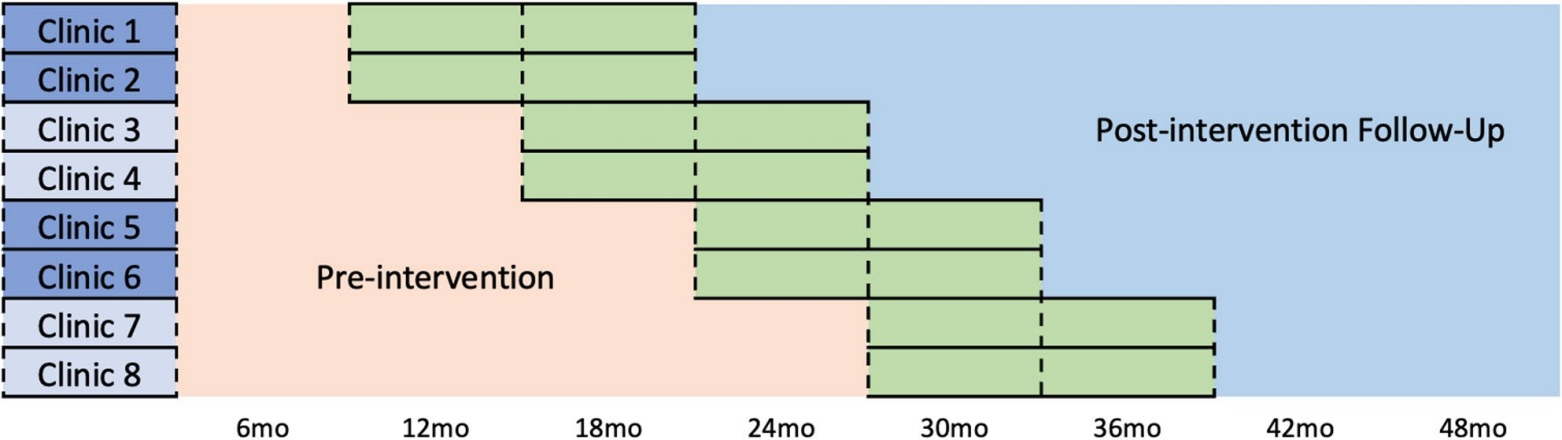
Project CASCADE stepped wedge design

We will randomize 8 CHC clinics in blocks of two every six months in a stepped-wedge design (Fig. 2). Each clinic team will implement the intervention over a 12-month period. The time before the intervention will serve as the usual care (control) condition, while the period during and after the intervention will constitute the treatment condition.

### Conceptual frameworks

**Fig. 3:**
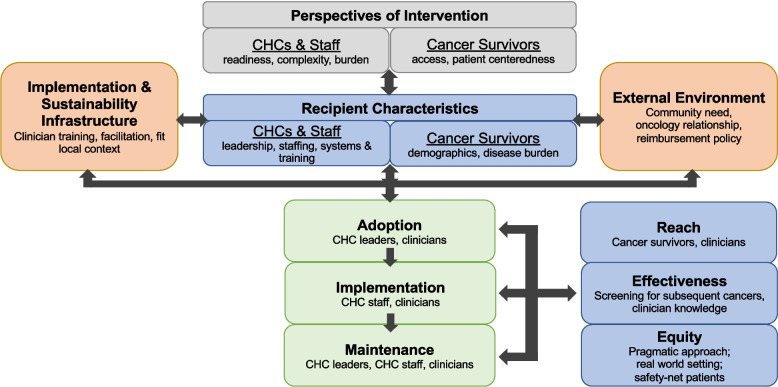
Use of PRISM within Project CASCADE

We will use the Practical, Robust, Implementation, and Sustainability Model (PRISM) [40] to guide the implementation of the intervention (Fig. 3). PRISM integrates key implementation and care delivery theories to enhance program implementation and sustainability. It identifies four key domains influencing intervention adoption and long-term success: intervention perspectives (usability, adaptability, and organizational readiness), recipient characteristics (leadership, staffing, patient demographics), external environment (policies, financial incentives, community resources), and implementation infrastructure (staffing, protocols, and support systems). PRISM emphasizes adaptability while addressing barriers to effective and equitable healthcare implementation. Figure 3 illustrates how PRISM is applied within Project CASCADE, highlighting the key contextual domains shaping intervention adoption, execution, and long-term impact.

**Fig. 4:**
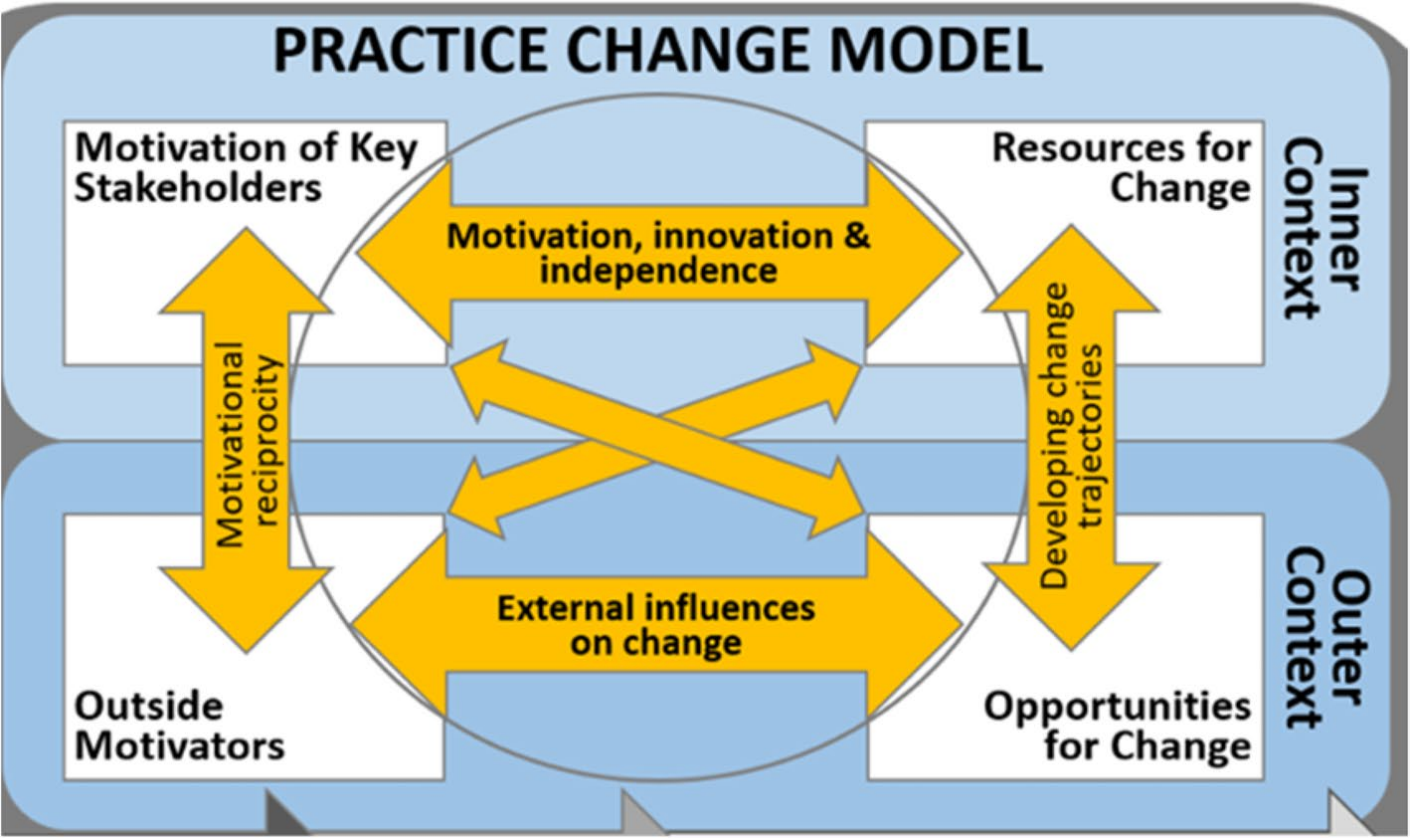
Practice change model

We will use the Primary Care Model (PCM) [41] to guide the evaluation of Project CASCADE. The PCM is empirically derived from a primary care-based preventive services intervention and envisions primary care clinics as complex, adaptive systems (Fig. 4) [41–43]. The PCM complements PRISM by assessing the motivation of clinic stakeholders, resources for change, opportunities clinics have to engage in change, and the effect of clinic’s external environment.

PRISM and PCM are both highly pragmatic, highlighting interaction of external and internal determinants to maximize learning from real-world settings.

### Intervention

The intervention will include three evidence-based components: (1) Training to enhance PCC knowledge of cancer survivorship care; (2) Tracking cancer survivors; and (3) Coordinating survivors’ care (Table 2). We used the forms/functions model to inform the development of the intervention [44]. Through this approach, we focus on intervention functions (the purpose of intervention components) and will then develop context-specific intervention forms for implementation to enhance intervention fidelity, adoption, and sustainment [40].

**Table 2:** Intervention components, functions, and anticipated forms

Barrier	Component	Expected Form(s)	Target
Lack of knowledge of cancer survivorship and follow-up	**Training** Enhance PCC knowledge of survivorship care	Provider-to-provider tele-mentoring	Clinic leaders and clinic team members
Insufficient attention to past history of cancer during primary care visits Uncertainty in primary care roles and responsibilities Lack of communication between Oncology and Primary Care	**Tracking** - Systematically identify cancer survivors - Monitor care delivery processes	Workflow modification Clinician reminders Survivorship care resources, e.g., Mobile apps recommended by the American Cancer Society	Clinicians and clinic staff
	**Coordinating** Designate a care coordinator champion	Designated personnel: e.g., MA, RN, or social worker to fit CHC context	Clinic staff, oncology partners, and cancer survivors

Training to enhance PCC knowledge of cancer survivorship care will include provider-to-provider tele-mentoring. Tele-mentoring sessions foster problem-solving through live dialogue between cancer experts and CHC teams to integrate survivorship care into routine practice [45–47]. These 1-h collaborative learning sessions will include didactic training and case-based learning using real patient cases identified by CHC teams. Clinic leaders will identify team members to participate in sessions, identify cases for discussion, and determine the schedule for the monthly sessions. Following each session, we will administer a brief survey to assess change in knowledge and attitudes regarding session content.

Tracking will include systematically assessing cancer history and monitoring survivors’ care to plan and organize subsequent care. Trained practice facilitators will work closely with clinic members to assess current workflows, staff roles and responsibilities, and existing chronic disease tracking processes. We will co-create protocols to assess cancer history using quality improvement (QI) tools. The clinic team may also create a cancer survivor registry or explore available EHR tools to aid in identifying and tracking survivors [48].

Coordinating requires each clinic leader to designate at least one staff member to be the care coordinator champion [49, 50]. Coordinator tasks will include gathering records, referring for appropriate screening, following up on results [39], and assisting patients in self-management [49, 51, 52]. Clinic leaders will determine task responsibilities, requisite competencies and supplemental training needs, practical frequency of contact, and how to monitor or measure performance. The clinic team will co-design, with practice facilitator support, survivorship resources at point of care, workflows operationalizing coordination pathways, and supporting patient outreach, awareness, and education. Monitoring may occur by telephone consultation or during in-person visits, either supplemental or adjacent to routine PCP visits [53–55].

### Implementation strategies

Two strategies (partner engagement and practice facilitation) will support intervention. Our evaluation will identify other emergent strategies and delineate mechanisms of implementation (Aim 3).

#### Partner engagement

FQHC Board of Directors composition must be 51% patients and include community members reflecting demographics of the service area [56–58]. Upon each clinic’s enrollment, clinic leaders will work with us to convene representatives of their Board, including at least one patient/client, and clinic staff designated by leaders (e.g., clinic manager). We will use group participatory approaches [59–61], drawing on participatory research principles [62–65], to engage partners in discussing challenges to delivering survivorship care in their specific setting. These techniques elicit cross-cutting perspectives grounded in the lived experience of each partner, generate insights from the local context, foster buy-in and accelerate group cohesion [66]. Eliciting these insights help establish shared goals of shaping clinic workflows to local context, thereby, increasing likelihood of the clinic’s ability to change [42]. The retreat will have three sessions: The Mission and Vision: Summarize latest evidence on cancer survivorship, present an environmental scan of oncology practices in CHC community (generated collaboratively by clinic teams and investigators) and brainstorm ways to partner with oncology representatives, and generate discussion about value of strong primary care-oncology partnerships; The Journey: Invite oncology and primary care partners to explore objectives for and challenges to care coordination focusing on concepts of shared leadership, [67–70] defined roles and responsibilities [71], then to brainstorm workflow changes that facilitate care for cancer survivors [72]; and The Tools: Present intervention functions and explore clinic-specific implementation of intervention forms, i.e., survivor identification and monitoring, the coordination role, and clinic workflow adaptation. Our clinic leaders are motivated to “dive in” for this collaborative approach to survivorship care. After each session, to stimulate discussion and solicit opinions to guide course correction, we will field a short, five-minute pragmatic survey modeled on existing measures, [73–75] to monitor acceptability, appropriateness, and feasibility, as real-time audit and feedback. Following the retreat, we will convene each clinic group quarterly to provide updates, maintain momentum, and resolve emergent challenges to ensure continued engagement of partners throughout implementation. Flowing from the retreat, we will meet with clinic teams over a three-month period to map components [76] to adapt workflows [77] to tailor implementation.

#### Practice facilitation

We will support each clinic’s intervention implementation by providing facilitation in quality improvement (QI) by a trained facilitator [78]. Facilitators will employ strategies to promote QI activities while also building capacity, [79] while strengthening our relationship with each CHC and team. Strategies include building relationships within teams, providing value by contributing EHR expertise, and remaining flexible throughout the QI change effort [80]. Facilitators will determine each clinic team’s readiness for change using the Organizational Readiness for Change measure [81] and will identify current needs and “pain points” [82]. While we have clinic leadership buy-in, it is critical to also cultivate buy-in from team members on the ground to promote intervention adoption. The facilitator will work with clinic teams in designing intervention forms that will achieve the tracking and coordinating functions. For example, the facilitator will support the designated care coordinator in planning and testing the process of assessing and monitoring survivors’ survivorship care needs. They will utilize established techniques such as PDSA cycles [83], clarifying roles and identifying champions [50], building trust through experience-based problem solving [84], workflow modification via cognitive walk-throughs [85, 86].

### Intervention rollout

The intervention will be implemented in each clinic over 12 months. Tele-mentoring sessions will start after the partners’ retreat for two clinics randomized to each block of the stepped-wedge design (Fig. 2). Tracking and coordinating components will “go live” during the week following the first tele-mentoring session. Modified workflows will assess patients for a history of cancer, at or before routine visits (e.g., through patient portal or via phone call). Each week, the “care coordinator” will review records for indication of cancer history, document cancer history in the problem list using a standardized format developed by the team, request records from oncology providers as needed, and pend to patient record with notification to PCC. We expect that PCCs will apply survivorship care knowledge gleaned from tele-mentoring sessions to plan survivors’ ongoing care. For patients who have finished cancer treatment, the coordinator will review treatment summary and care plan upon receipt and assess survivorship care needs (e.g., screening for second primary cancer, adherence to recommended cancer surveillance). For patients in active cancer treatment, the coordinator will facilitate communication [87] with oncology about appointments, treatments, and potential impact on chronic disease care, and encourage continued connection with primary care [88–90]. The coordinator will review with each patient roles and responsibilities of respective providers, advise on appropriate follow-up, and encourage continual interaction with their primary care team. They will also ensure patients receive relevant referrals to smoking cessation, diet and exercise, and psychosocial counseling, as appropriate. Information and education will be culturally and literacy-level appropriate. Practice facilitators will set up monthly in-person or web meetings to help the clinic team reflect [86] and discuss how to identify survivors, clarify team member roles, foster communication, manage care transitions, and monitor or adjust workflow [91–94].

### Outcomes

The primary outcome for this study is the proportion of patients who have received guideline-concordant screening to detect second primary cancers (i.e., breast, cervical, colorectal, lung, and prostate cancer screening for which there are established clinical NCCN guidelines). This outcome is an NCCN guideline [95], is clinically significant, and relevant for CHCs [96]. Cancer survivors have higher risk for second cancers, so increasing screening aids early detection and treatment, reducing morbidity and mortality [95, 97]. Moreover, this outcome is pragmatic, falling within the scope of primary care practice and pertinent to CHCs annual Uniform Data System (UDS) performance metrics [98]. We hypothesize that compared to usual care periods, post-intervention periods will have higher rates of cancer screening for second primary cancers among cancer survivors seen during routine primary care.

We will assess the following secondary outcomes:

1. Physician-reported cancer survivorship care knowledge and skills. We hypothesize that in the post-intervention periods, physicians will report enhanced cancer survivorship care knowledge and skills compared to the usual care period. Provider knowledge and skills will be assessed by self-administered survey.
2. Experience of cancer survivors with care coordination. We hypothesize that patients will report better experience with care coordination post-intervention compared to usual care. Patients' experience with care coordination will be assessed via survey.
3. Adherence to NCCN surveillance guidelines for breast, cervical, colorectal, lung and prostate cancer. We hypothesize that post-intervention periods will have higher rates of guideline-concordant cancer surveillance compared to usual care periods [99–103].

### Quantitative data collection

#### EHR extraction/audit

The study team with support from clinic EHR specialists will extract EHR data for eligible patients at the end of every 6-month period. Extracts will include all patients with a history of cancer who were seen in the respective 6-month period.

#### Clinic survey

The clinic manager/lead clinician will complete a Clinic Survey capturing structural (type, size, number of FTE clinicians, patient panel demographics, payer sources), operational (use of QI strategies such as team huddles and audit & feedback), and EHR characteristics. We will use REDCap to create the survey, and field data collectors will distribute QR codes to all clinic members to complete it anonymously.

#### Clinic member survey

The Clinic Member Survey will include adapted SPARCCS survey items (for clinicians) and clinic readiness, capacity to implement change, leadership, implementation climate and culture, teamwork, and team communication (for all clinic members)—attributes associated with implementation success [41, 42, 104–109].

#### Cost surveys

Measure cost to clinics of implementing the intervention. Clinic managers will complete a survey once every year during the intervention providing personnel time, salary information, cost of materials, and medical care costs related to intervention start-up: (1) personnel effort, software, hardware and costs related to workflow modification, (2) time and effort assigning coordinator responsibilities and identifying eligible patients, (3) tele-mentoring delivery and participation, and intervention execution costs including: (1) effort of staff assigned to care coordinator role, workflow modification(s), cancer documentation, EHR tool maintenance as applicable, and (2) healthcare utilization costs for survivors receiving the intervention.

### Qualitative data collection

To guide qualitative data collection, we will use the Rapid Assessment Procedure Informed Clinical Ethnography (RAPICE), a method that uses trained qualitative fieldworkers for participant observation and interviewing [110]. We will use RAPICE to guide our collection of observational and qualitative data before, during, and after implementation.

#### Observations

We will also conduct direct observations of tele-mentoring sessions and clinic activities using a previously established observation template [111]. Observations will focus on clinic locations (e.g., waiting room, nursing area), circumstances (e.g., walk-ins, late arrivals), and visit type (e.g., new patient, annual, follow-up, survivorship care) to gain insight on how survivorship care is delivered. Facilitators will take short notes, later expanding them into full fieldnotes supplemented by existing documents such as flow sheets, schedules, protocols, and annual reports.

#### Clinic member interviews

We will contact clinics to set up site-visit logistics and to obtain up-to-date lists of clinic members. We will conduct in-depth, one-hour interviews with administrators and clinicians, care coordinators, and board members to elicit narratives about intervention experiences, feasibility, and sustainability. For example, clinician interviews will assess how survivorship care training and facilitated tracking and coordinating supports ongoing care management of survivors.

#### Patient interviews

We will conduct patient interviews to elucidate experiences of care coordination and PCM domains: attitudes toward follow-up care; competing demands; and comprehension of clinic communications [40]. We will purposively sample patients to compare between clinics. Patient Surveys will measure the *Picker Patient Experience of Care care coordination subscale* and other potential confounders and effect modifiers [112, 113]. We. Surveys will be administered by phone during the pre- and then again during the post-intervention period. We have already translated these survey measures in Spanish, made them culturally appropriate, and understandable by individuals with low literacy (*R01CA203856*)*.*

### Data analysis

Initially we will examine the distribution of each variable. We will then check for out-of-range values, outliers and abnormal values using graphical methods such as the box-plot. We will verify that the distributions of measures meet the assumptions of the statistical tests to be used, applying a formal test such as the Shapiro–Wilk’s test [114]. If the assumptions are not fulfilled, we will consider appropriate transformations or application of alternative methods such as non-parametric inference. Further, we will test whether baseline characteristics are well balanced between groups. If a baseline variable is not balanced, we will include it as a covariate in the analysis. To test the superiority of the intervention compared to the control (usual care) we will adopt the linear mixed-effect (LME) model for a cross-sectional stepped-wedge design. In this design it is assumed that the analysis will include data from subjects both before and after the crossover, with subjects distributed proportionately across the trial timeline. Our basic model is a mixed-effect regression model where the outcome of interest is the proportion of cancer survivors screened for other cancer types. The outcome will be transformed in the normal scale using a logistic transformation before fitting LME if needed. The model will include a cluster/clinic-specific random effect, fixed effects due to time/period, and an intervention indicator variable taking on the value “1” if the cluster is given the active intervention and “0” otherwise. As the assignment of cluster changes, so does the value of this indicator variable with time. Our primary aim is to test the significance of the intervention effect associated with the intervention indicator variable. The alternative model includes Heo et al. (2018), which argued that the time/period effect could be considered a random period-level model nested within the cluster level, resulting in a slightly different sample size [115]. For our primary outcome, we plan to use Hussey and Hughes’s (2007) model with the time/period effect as a fixed effect [34].

#### Power calculation

The primary outcome variable is continuous, for which Hussey and Hughes (2007) derived a closed-form analytical expression for power using linear mixed-effect model [34]. This formula is implemented in the PASS 2022 software, which is used for power calculation. From the historical records of recruitment feasibility, we propose to recruit 100 subjects (or more) per clinic in every six-month period. This results in a sample size N = 100*8*6 = 4800, out of which about 2000 will be under usual care and 2800 under intervention condition (excluding intervention implementation period in each clinic). We assume an ICC < 0.05 (within clinic association). We assume the difference in screening rate between intervention and usual care condition is ≥0.15 to guarantee ≥85% power, with a two-sided type-1 error of 0.05 (see power curve figure). Sample size using Heo et al. (2018) is slightly larger but still guarantees ≥ 80% power (checked using the swCRTdesign package in R software) [115]. A cross-sectional stepped wedge design assumes that outcomes are assessed for unique survivors every six months, and we recognize that eligible patients may return for regular clinic visits. However, for any eligible survivor, if the outcome of cancer screening in the past period is negative, then that past outcome does not affect the outcome of screening in the future period, and survivors who screened positive (in any period) are then not eligible for outcome assessment in future periods. The stepped-wedge design can be implemented in two forms: cross-sectional and repeated-measures. We have chosen a cross-sectional stepped-wedge design (open cohort), which assumes that clinics may evaluate new subjects during each observation window (i.e., every six months). While it is possible that a subject who screened negative in a previous period may return for screening in a later period (if still eligible), we expect the number of such repeat participants to be low. Importantly, for any given subject, a negative screening outcome in a previous period does not influence the likelihood of a positive outcome in a subsequent period. Subjects who screen positive in any period are referred for appropriate treatment and removed from future screening. Therefore, no sample size adjustment has been made for potential repeated observations within a clinic. Missing data is minimal in this cross-sectional design due to long-standing CHC relationships and ongoing partner engagement.

#### Secondary outcomes

We will conduct a cluster-adjusted two-sample t-test and/or mixed effect model to compare usual care and post-intervention outcomes. We will use a group (pre-/post- intervention) indicator as a fixed effect and a clinic-specific indicator as a random effect. We will also use a time-specific fixed effect. We will include a group*time interaction term to identify group-specific differential change in the secondary outcome between usual care and post-intervention time periods.

We will use graphical methods to examine variable distribution for outliers and abnormal values and verify statistical test assumptions using formal tests (e.g., Shapiro Wilks). We will utilize transformations or non-parametric methods to address any unmet assumptions. The clinic will serve as the unit of analysis. All tests will have a two-tailed alpha level of 0.05.

#### Mixed-method evaluation

Qualitative data collection and analysis will be iterative; as our understanding of clinic factors enhancing care of survivors expands, additional areas for exploration may emerge. The initial stage will entail an intensive series of analysis and interpretive steps with potential for additional data collection to generate summaries of individual clinics. We will treat each clinic as a separate case in its own context, integrating relevant quantitative data into each case. Each case provides a preliminary interpretation of collected data, triangulating findings from multiple data sources (surveys, interviews, documents, clinical EHR data) using PCM attributes [40]. In each case we examine the role of PCM determinants. For example:

- *How do clinic team motivations affect implementation processes and success?*
- *How do clinic-level structures, workflows, and referral processes (resources for change) facilitate or impede implementation?*
- *How do local, state and federal policies (outside motivators) affect the motivation of clinics, providers and other stakeholders to implement intervention (motivational reciprocity)?*

The team will update a working summary of emergent findings and iteratively query facilitators as needed to gain insight into the data, their context, and additional data collection targets until we reach saturation. Points of convergence and divergence will be discussed with the research team until consensus is achieved. If consensus is not achieved, we will conduct follow-up interviews to resolve interpretation divergence. Lastly, interpretation of findings will be member-checked with clinic partners to confirm validity.

#### Cross-case synthesis

Cross-case comparative analysis will be conducted, first by generating interpretive summaries using mixed-method matrices to display data across case studies comparing individual sites [116, 117], then identifying overarching insights emphasizing core features that promote implementation success. We will integrate quantitative data on intervention and implementation outcomes into each qualitative case study to generate comprehensive understanding of clinic-level implementation. We use the five phases of qualitative interpretive analysis [118, 119], which include describing, organizing, connecting, corroborating, and representing the account. This five-step process results in interpretive summaries addressing implementation research questions. For example, to address RQ1, we will integrate clinic member survey data on feasibility, acceptability, and appropriateness of intervention with themes from provider interviews and clinic observations, first for each clinic and then across clinics. We will assess fidelity considering degree to which intervention components are implemented as planned and extent components or attendant processes were adjusted to fit local settings [120, 121]. Key questions include: How do team relationships create challenges and opportunities, e.g. “workarounds”, to improve care coordination for survivors? We will employ best practices for analyzing mixed methods data, including member checking and triangulation of multiple data sources, and engaging multiple investigators to review interpretations and control for bias [122]. We will share final summaries and visual presentations with CHC partners. Importantly, we will synthesize cross-case findings into a step-by-step implementation guide for dissemination and scale-up to other health systems [123].

#### Cost and cost-effectiveness analyses

We will estimate the incremental cost effectiveness ratio (ICER) [124, 125], which is the incremental cost of the intervention compared to usual care divided by the incremental difference in the proportion of patients receiving guideline appropriate care. The 95% confidence interval for the ICER assesses uncertainty through a nonparametric bootstrapping approach [126]. If the ICER replicates cover more than one quadrant of the cost-effectiveness plane, the cost-effectiveness acceptability curve approach will be used to capture the uncertainty [127–129]. We will conduct sensitivity analyses to determine the robustness of change in values of the main model parameters on ICER estimates.

#### Data sharing procedure

The de-identified data final datasets, with any necessary study identifiers (excluding those prohibited by HIPAA) may be made available to other UTHealth, KUMC, UT Southwestern, and MDA investigators, as well as the external investigators following appropriate approvals and processes through the openICPSR.

#### Ethical considerations

This study was approved by the Committee for the Protection of Human Subjects at the University of Texas Health Science Center at Houston with a full waiver of consent for EHR data extraction and a waiver of written informed consent for surveys and interviews. All data will be de-identified upon receipt by the study team and identifiable information will be stored separately from the analytic dataset on password-protected servers both within the study team and the clinic of origin. All personnel interacting with participants and having access to data will be trained in human subject protections and confidentiality procedures.

## Discussion

Project CASCADE tests a “generalist” primary care-based care delivery intervention that integrates cancer survivorship care with routine primary care for all conditions. It focuses on minority, under- and uninsured survivors in CHCs who experience significantly higher burden of poor outcomes, addressing health equity concerns [130]. This study engages CHC clinicians, patients, and community representatives throughout the study, with the aim of increasing adoption and sustainability [56]. By collaborating with CHCs, it has the potential to advance health equity by bringing care delivery innovations to patients most likely to experience poor health outcomes [131].

One third of CHC cancer survivors are racial/ethnic minorities; about two thirds are under- or uninsured [28]. These survivors also have co-occurring conditions: 20% of breast cancer survivors have type II diabetes and 43% of colorectal cancer survivors have hypertension [28]. Suboptimal chronic disease management among cancer survivors also negatively impacts survival [132, 133]. Compounding social and medical risks, health system barriers (e.g., access, financial coverage, communication between providers) disproportionally impact low income, Black, and Hispanic cancer survivors [134–139]. Fragmented cancer and chronic disease care and suboptimal communication between primary care and oncology disproportionally impact underserved survivors, who suffer greater morbidity and mortality from both cancer and chronic illnesses [88]. Research on how to deliver coordinated, comprehensive care is critical for improving health outcomes for such underserved individuals [56]. Our study seeks to test ways to integrate survivorship care delivery innovations into routine primary care in these community clinical settings [20, 27].

This study pairs the hybrid implementation-effectiveness approach [140, 141] with an iterative, mixed-methods evaluation that will elucidate *why* and *how* the intervention worked or did not, thus generating actionable findings for dissemination, transportability, and scalability to other settings [123]. These methods advance pragmatic implementation science for healthcare delivery research [142].

In partnership with CHC partners, the study will generate the evidence-base needed for a generalist primary care-based care delivery model with the goal of disseminating and scaling to optimize cancer survivorship care equitably. If effective, the study will also enhance the capacity of safety-net primary care teams to manage cancer survivorship in primary care, enabling survivors cared for by CHCs to receive coordinated, whole-person care.

### Strengths and limitations

This study presents several strengths that make it both innovative and impactful. By working directly with CHCs, Project CASCADE will advance health equity and address care disparities in underserved populations. The participatory and pragmatic approach not only enhances the relevance and responsiveness of the intervention but also increases its likelihood of adoption and long-term sustainability within CHCs. Additionally, the study evaluates the impact of the intervention at multiple levels—clinician, patient, and system — allowing for a comprehensive understanding of both effectiveness and implementation.

The study also has limitations worth noting. Engaging clinics in research can be challenging; however, in our experience, clinics and stakeholders remain more engaged when researchers seek and incorporate their input from the outset. Additionally, implementing practice changes can be burdensome in busy practices; However, this pragmatic intervention was designed to fit closely with scope of primary care practice. Finally, accurate identification of patients with a history of cancer is challenging, raising the risk of misclassification bias. Inconsistent documentation practices or missing historical data within EHRs may lead to underestimation or misidentification of eligible patients but plans for clinic workflow redesign with CHC partners targets these historical inconsistencies.

## Conclusion

Project CASCADE offers a scalable, evidence-based approach to integrating cancer survivorship care into primary care, particularly within safety-net settings. By leveraging a pragmatic, participatory design, this study will enhance the capacity of CHCs to deliver equitable and comprehensive care for cancer survivors. Findings will inform future efforts to optimize survivorship care delivery, ensuring that primary care teams can effectively manage the long-term needs of this growing patient population.

## Supplementary Information

Supplementary Material 1.

## Data Availability

No datasets were generated or analysed during the current study.

## Abbreviations

CHC: Community health center
CONSORT: Consolidated Standards of Reporting Trials
HER: Electronic health record
FQHC: Federally qualified health center
PCC: Primary care clinician
PCM: Practice change model
PRISM: Practical, Robust, Implementation, and Sustainability Model
QI: Quality improvement
RAPICE: Rapid Assessment Procedure Informed Clinical Ethnography
SPARCCS: Survey of primary care physician attitudes regarding the care of cancer survivors
SPIRIT: Standard Protocol Items: Recommendations for Interventional Trials
StaRI: Standards for Reporting Implementation Studies
US: United States
USPSTF: U.S. Preventive Services Task Force
